# The Importance of Gender of Patients and General Practitioners in Relation to Treatment Practices for Overweight

**DOI:** 10.1371/journal.pone.0095706

**Published:** 2014-04-22

**Authors:** Jeanett Friis Rohde, Marie Vik Hessner, Jørgen Lous, Pia Müller, Ulla Hølund, Berit Lilienthal Heitmann

**Affiliations:** 1 Institute of Preventive Medicine, Bispebjerg and Frederiksberg Hospital, The Capital Region, Copenhagen, Denmark; 2 Institute of Public Health, Research Unit of General Practice, University of Southern Denmark, Odense, Denmark; 3 Institute of Public Health, Section of General Practice, University of Copenhagen, Copenhagen, Denmark; 4 Danish Health and Medicines Authority, Copenhagen, Denmark; 5 The Boden Institute of Obesity, Nutrition Exercise & Eating Disorders, University of Sydney, Sydney, Australia; 6 National Institute of Public Health, University of Southern Denmark, Copenhagen, Denmark; Faculty of Medicine & Allied Sciences, Rajarata Univeresity of Sri Lanka, Sri Lanka

## Abstract

**Background:**

Several studies suggest that men and women are treated differently for similar disease including diabetes and cardiovascular disease. Differences in attitudes and treatment practices towards men and women with obesity are not well recognized.

**Objective:**

To investigate the attitudes and treatment practices among Danish general practitioners (GPs), in relation to treatment of overweight, while taking gender of both the patients and practitioners into account.

**Design:**

Questionnaire inventory covertly examining attitudes and practices among Danish general practitioners towards treatment of overweight. All 3.637 general practitioners from the Danish Medical Association register were invited to participate in the survey. In total 1.136 participated.

**Results:**

The GPs found weight loss to be more important for overweight male than overweight female patients. They also treated complications to overweight more rigorously among male than female patients, and recommended lipid lowering medicine more often to male than female overweight patients. In addition, the younger female GPs and older male GPs more often said that they would treat an overweight patient with lipid lowering medicine.

**Conclusion:**

Among general practitioners in Denmark, treatment for weight loss is more often practiced for overweight male than overweight female patients presenting with same symptoms. In addition, hyperlipidemia among overweight males is also more often treated with lipid lowering medicine than hyperlipidemia among overweight females.

## Introduction

A number of studies have shown that the relative risk of health complications related to obesity seems more severe for women than men [1]. Incidence of diabetes, for instance, is two- to four-fold higher for overweight and obese women than men [2], [3], and risk of cardiovascular disease is twice as high among women than men with type 2 diabetes [4], which causes diabetic women’s absolute risk of CVD to approach that of men with diabetes [5]. Absolute incidence and death from CVD is clearly overall more prevalent in men than in women, at least until age 70, but the relative risk of CVD related to obesity is greater for women than men [6], and female CVD patients have been shown to have a worse prognosis compared to male CVD patients [7], [8]. Also, earlier studies by Larsson et al [9], [10] suggested that for the same degree of abdominal obesity, the risk of CVD was similar for men and women, while a more recent Polish study found that women with abdominal obesity suffered more often from CVD than men with abdominal obesity [11]. Furthermore, a recent Danish study found that for the same degree of obesity, women had more mental and physical health problems, including depression, fertility problems, and osteoarthritis [12], and an American study showed that women with obesity suffer more from stress, headache, tiredness and depression than men with obesity [13]. The higher relative morbidity associated with female compared to male obesity suggests that overweight and obesity may have to be taken more seriously by health professionals when occurring among women than among men. In numerous studies however, compared to men, women have been found to receive suboptimal CVD preventive care [7], they are screened and treated less aggressively for their illnesses, or are even less likely to undergo cardiac procedures [8]. Furthermore, one study found that among Swedish general practitioners, treatment for obesity was offered sooner to obese men than to obese women, but paradoxically, more often assistance with weight loss was given to normal weight women than normal weight men, despite the obvious lack of need for such treatment [14]. A review by Budd et al. [15] about changes in attitudes and beliefs towards obesity of health care professionals showed that although primarily still negative, attitudes and beliefs of the health care professionals have improved over time and do not generally any longer have a negative effect on the delivery of care, except for possibly among younger health care professionals [15].

We showed in an earlier study that in relation to preventing CVD, Danish general practitioners would more often recommend diet counseling and weight loss to overweight male than female patients presenting with similar symptoms. Then we suggested that health professionals need to appreciate this treatment bias and increase their awareness in order to deliver prevention and treatment strategies that take the gender of patients into account [16]. This earlier study was conducted 15 years ago, and the medical education system, as well as the practices and attitudes among GPs will most likely have changed since then, as also suggested by Budd et al. [15]. Therefore, we examined present attitudes and practices among all Danish general practitioners of the new millennium, with regard to prevention and treatment of overweight and its complications, while taking gender of both patients and practitioners, and age of the practitioners, into account.

## Method

The present questionnaire survey examined attitudes and practices among Danish general practitioners (GPs) towards prevention and treatment of overweight among female and male patients. The study questionnaire that was sent out to all Danish GPs was a replication of a previous survey from 1994 [16]. The present study was conducted in 2008. The participating GPs were all from the Danish Medical Association register, and the questionnaire was sent to a total of 3.637 GPs in Denmark, including those in Greenland and The Faroe Islands. In total, 1.151 returned the questionnaire. No answers were received from Greenland, and only four answers were received from the Faroe Islands. These were subsequently excluded from the analyses. Moreover, two people were excluded because of missing information regarding the Region they were practicing in, and nine GPs were excluded because of missing information about their gender. The total number of GPs included in the analysis was 1.136.

The GPs received a 12-page questionnaire, which, in addition to a number of general questions on attitudes, included ten case reports. In the general part of the questionnaire, the GPs were asked to state their gender and age. They were also asked how many GPs were working in the practice and the number of patients associated with their practice. Moreover, they were asked about the number of patients consulted pr. day.

The GPs were furthermore asked if they advise women more frequently than men about weight loss, and if they believe that men and women should have the same advice in relation to prevention of obesity. Answering possibilities included: mainly agree, mainly disagree, or neither agree nor disagree.

Finally, the questionnaire included ten case reports about CVD, cancer, osteoporosis and under/overweight. Half of the GPs received case reports that concerned female patients and the other half received reports that concerned male patients. Apart from gender, case reports were similar for content. The GPs were not informed that there were differences in the genders of the case reports, and male and female cases were allocated at random to the GPs. In the present paper, we have chosen to look further into the answers given in relation to the one case report that specifically concerned an overweight male or female patient. In total, 984 of the GPs returned answers to the case report with full details.

The female and male case report read as follows:

*A 50* *year old healthy, postmenopausal woman. Slightly overweight. Non-smoker. No cardiovascular disease in the family. Normal blood pressure. She intended to get her cholesterol measured, which turned out to be 8.0* *mmol/l. triglycerides 1.7* *mmol/l, HDL-cholesterol 1.0* *mmol/l.*

*or*

*A 50* *year old healthy man. Slightly overweight. Non-smoker. No cardiovascular disease in the family. Normal blood pressure. He intended to get his cholesterol measured, which turned out to be 8.0* *mmol/L, triglycerides 1.7* *mmol/l, HDL cholesterol 1.0* *mmol/l.*

For each specific case report, the GPs were asked their opinion about recommending lipid lowering medicine, exercise, weight loss, dietary counseling, or unchanged lifestyle as treatment. The response options included: very important, important, less important or not important. In our analyses we coded responses into two categories: very important/important, and less important/not important.

### Ethical Considerations

The GPs received a questionnaire and an introduction letter describing the purpose of the study. They were informed that their answers were anonymous and therefore, as there were no ethical considerations, we did not obtain informed consent.

### Statistical Analysis

We used chi-squared to test the attitudes among GPs towards prevention, e.g. if male and female patients should have the same advice about prevention of obesity, or if the GPs advised overweight female patients more often than overweight male patients about weight loss. Logistic regression was used for the analysis of the case reports, and gender of patients and GPs, as well as GPs age, were included as covariates. Before running the final logistic regression analyses, we tested for interactions. More specifically, we tested whether the higher odds for recommending weight loss and lipid lowering medicine to female than male overweight patients was influenced by gender and age of the GPs. We found an interaction between age and gender of the GP, and lower odds for recommending lipids to overweight female than male patients (p = 0.049). Because of this interaction between GPs gender and age in the analysis concerning lipid lowering medicine, further analyses were stratified by gender of the GP. Furthermore, a binary variable for the age of the GPs was created from the continuous age variable and was divided by the 50% percentile for male and female GPs. Differences were considered significant at p<0.05. The statistical analyses where performed with Stata 9.0 programme.

## Results

In total, 3.637 GPs were invited to participate in the survey and 1.151 returned the questionnaire. Of them, 15 GPs were excluded due to different reasons mentioned earlier giving an overall response rate of 31%. In total, 984/1151 (87%) of the GPs gave complete answers to each of the five treatment strategies.

Among the 1.136 included GPs, 58.7% were male and 41.3% female. Data from the Danish Medical Association register showed that there were 62,1% male GPs and 37,9% female GPs registered in Denmark in 2008 [17]. Distribution of the GPs gender, age, practice type, region and size of practice by patient’s gender are given in table 1. The mean age for all GPs was 53 years, and the age distribution of the GPs was equally distributed between male and female cases (p = 0.47). Most GPs worked in practices as part of a team, and had between 1500 and 1999 patients assigned per GP (n = 551, table 1). Moreover, the results showed that there seemed to be a significant differences (p<0.001) in the allocation of which Region the GPs were coming from, when looking at male and female cases.

**Table 1:** Distribution of GPs gender, age, practice type, region and size of practice by patients gender.

	N	Female patients	Male patients	P (Male and female differences)
		Mean	Mean	
***Gender GP***	***1136***			*0.47*
*Female*	*469*	*40.3*	*42.4*	
*Male*	*667*	*59.7*	*57.6*	
***Age GP (Years)***	***1132***	*52.2*	*53.4*	*0.07*
***Practice type (%)***	***1134***			*0.61*
*Team practice*	*766*	*68.2*	*66.8*	
*Solo practice*	*368*	*31.8*	*33.2*	
***Region (%)***	***1123***			*<0.001*
*Capital*	*363*	*29.1*	*36.0*	
*Zealand*	*179*	*19.3*	*12.1*	
*South Denmark*	*238*	*16.0*	*27.1*	
*Central Denmark*	*231*	*23.0*	*17.8*	
*North Denmark*	*112*	*12.6*	*7.0*	
***Patients assigned (%)***	***1132***			*0.95*
*>500*	*2*	*0.2*	*0.2*	
*500–999*	*26*	*2.5*	*2.1*	
*1000–1499*	*340*	*29.5*	*30.7*	
*1500–1999*	*551*	*48.1*	*49.3*	
*2000–2499*	*90*	*8.3*	*7.5*	
*<2500*	*123*	*11.5*	*10.2*	

The total number GPs may not be 1136 in all categories because of missing data.

Table 2 showed the GPs answers regarding practices towards treatment and prevention. To the question: “I believe that men and women should have the same advice in relation to prevention of obesity”, almost all (92.3%) of the GPs stated that they mainly agreed, and no answering differences were seen between male and female GPs (p = 0.47). In total, 56.9% of the GPs stated that they mainly agreed that they more often advised women than men about weight loss. There was a significant difference in how male and female GPs answered this question, and more female (64.3%) than male (49.5%) GPs stated that they more often advised female than male patients about weight loss (p<0.001).

**Table 2:** GPs answers regarding practices towards treatment and prevention.

	N	Female (%)	Male (%)	p
***Q1*** ** “** ***I advise women more frequently than men about weight loss*** **”.**				
*Total*	***1131***			
*Mainly agree*	*629*	*64.3*	*49.5*	*<0.001*
*Mainly disagree*	*303*	*22.0*	*30.2*	
*Neither agree nor disagree*	*199*	*13.7*	*20.4*	
***Q2 “I believe that men and women should have the same*** ***advise in relation to prevention of overweight”.***				
*Total*	***1122***			
*Mainly agree*	*1036*	*91.6*	*92.9*	*0.47*
*Mainly disagree*	*51*	*4.5*	*4.6*	
*Neither agree nor disagree*	*35*	*3.9*	*2.6*	

The total number GPs may not be 1136 in all categories because of missing data.

Table 3 shows the distribution of GPs that found a specific treatment important/not important for male and female cases. Most of the GPs recommended diet counseling (97.0%) and exercise (97.0%) as treatments for overweight for both male and female cases. However, the results from the case reports (table 4) showed that irrespective of the gender or the age of the GP (p = 0.22), the odds for being advised to lose weight was lower for a female overweight patient than for a male overweight patient (OR = 0.62 (95% CI 0.48–0.80)). Irrespective of the gender of the GPs (p = 0.47), slightly younger than older GPs recommended weight loss to their patients (OR = 0.98 (95% CI 0.96–1.00)). Furthermore, in regards to pharmacological treatment with lipid lowering medicine, the odds for being advised to take such medicine was lower for the female than the male overweight patients (OR = 0.70 (95% CI 0.55–0.90)). In general, both male and female GPs more often recommended lipid lowering medicine to their male, than to their female patients (p = 0.01).

**Table 3:** Case report: Distribution of GP’s finding a specific treatment important/not important for male and female patients.

	N	N (%)	Female (%)	Male (%)	p
**Weight loss**					
*Total*	***1082***				*<0.001*
*Not important*	*388*	*35.9*	*40.8*	*30.3*	
*Important*	*694*	*64.1*	*59.2*	*69,7*	
**Diet**					
*Total*	***1105***				*0.86*
*Not important*	*33*	*3.0*	*2.9*	*3.1*	
*Important*	*1072*	*97.0*	*97.1*	*96.9*	
**Lipid lowering medicine**					
*Total*	***1063***				*0.01*
*Not important*	*435*	*40.9*	*44.7*	*36.7*	
*Important*	*628*	*59.1*	*55.3*	*63.3*	
**Exercise**					
*Total*	***1102***				*0.21*
*Not important*	*33*	*3.0*	*2.4*	*3.7*	
*Important*	*1069*	*97.0*	*97.6*	*96.3*	
**Unchanged lifestyle**					
*Total*	***984***				*0.99*
*Not important*	*916*	*93.1*	*93.1*	*93.1*	
*Important*	*68*	*6.1*	*6.9*	*6.9*	

**Table 4:** Case report: Odds and CI’s for GP’s advising weight loss or treatment with lipid lowering medicine to overweight patients.

	Weight loss			Lipid lowering medicine		
	N: 1078			N:1059		
	OR	CI	p	OR	CI	p
***Patient’s gender (female)***	*0.62*	*0.48–0.80*	*<0.001*	*0.70*	*0.55–0.90*	*0.01*
***GPs gender (female)***	*1.27*	*0.97–1.67*	*0.09*	*0.77*	*0.60–1.03*	*0.08*
***GP’s age***	*0.97*	*0.96–1.00*	*0.01*	*0.99*	*0.97–1.01*	*0.25*
***Test for interaction***			***p***			***p***
GPs gender*GPs age			*0.47*			*0.05*
GPs gender*Patient’s gender			*0.63*			*0.84*
Patient’s gender*GPs age			*0.22*			*0.64*

Note: Reference category: Male patients, Male GP’s. Values shown: Odds Ratio, CI and P-value.

Because of a significant interaction between GPs gender and age in the analysis related to treatment with lipid lowering medicine, all subsequent sub-analyses were categorized by gender of the GPs (table 5 and 6). These final analyses showed that both male (p = 0.04) and female GPs (p = 0.04) found lipid lowering medicine to be more important for male than female overweight cases (table 5). Moreover, younger female GPs seemed to recommend lipid lowering medicine more often than older female GPs (p = 0.02). In regards to recommending weight loss to overweight patients, both male (p = 0.01) and female GPs (0.01) found it more important for male than female patients. Furthermore younger female GPs found weight loss to be more important than older female GPs (p = 0.03) (table 6).

**Table 5:** Case report: Odds and CI’s for GPs advising treatment with lipid lowering medicine to overweight patients.

	Male GPs			Female GPs		
	N: 622			N:437		
	OR	CI	p	OR	CI	p
***Patient’s gender (female)***	*0.72*	*0.52–0.99*	*0.04*	*0.66*	*0.45–0.97*	*0.04*
***GP’s age***	*1.00*	*0.98–1.02*	*0.77*	*0.97*	*0.94–1.00*	*0.02*
***Test for interaction***						***p***
GPs gender*GPs age						*0.05*
GPs gender*Patient’s gender						*0.84*
Patient’s gender*GPs age						*0.64*

Note: Reference category: Male patients, Male GP’s. Values shown: Odds Ratio, CI and P-value.

**Table 6:** Case report: Odds and CI’s for GPs advising weight loss to overweight patients.

	Male GPs			Female GPs		
	N: 630			N:452		
	*OR*	*CI*	*p*	*OR*	*CI*	*p*
***Patient’s gender (female)***	*0.66*	*0.47–0.91*	*0.01*	*0.56*	*0.37–0.84*	*0.01*
***GP’s age***	*0.98*	*0.96–1.00*	*0.11*	*0.97*	*0.94–1.00*	*0.03*
***Test for interaction***						***p***
GPs gender*GPs age						*0.47*
GPs gender*Patient’s gender						*0.63*
Patient’s gender*GPs age						*0.22*

Note: Reference category: Male patients, Male GP’s. Values shown: Odds Ratio, CI and P-value.

Tables 7 and 8 show the fraction of overweight female and male patients for whom lipid lowering medicine and weight loss, respectively, was thought to be important/not important by age and gender of the GPs. Younger female GPs (≤48 years), found it more important to recommend lipid lowering medicine to the male (71.7%) than female (54.5%) overweight patients (p = 0.01). Moreover, older male GPs (≥57 years) found it more important to recommend lipid lowering medicine for male (69.6%) than female (56.3%) overweight patients (p = 0.01). Regarding weight loss, older female GPs (≥49 years) found it more important to recommend weight loss to overweight males (72.2%) than females (55.0%) (p = 0.01). Younger male GPs (≤56 years) found it more important to recommend weight loss to overweight male (71.4%) than female (54.8%) patients (p = 0.004).

**Table 7:** Case report: Fraction of overweight female and male patients for whom it was thought to be important/not important to use lipid lowering medicine by age and gender of GPs.

	Lipid lowering medicine				
GPs age	N	Patients gender	Not important	Important	p
**≥57 years (male GPs)**	*328*				*0.01*
	*161*	*Male*	*30.4*	*69.6*	
	*167*	*Female*	*43.7*	*56.3*	
**≤56 years (male GPs)**	*294*				*0.79*
	*128*	*Male*	*40.6*	*59.4*	
	*166*	*Female*	*42.2*	*57.8*	
**≥49 years (female GPs)**	*222*				*0.61*
	*119*	*Male*	*47.1*	*52.9*	
	*103*	*Female*	*50.5*	*49.5*	
**≤48 years (female GPs)**	*215*				*0.01*
	*92*	*Male*	*28.3*	*71.7*	
	*123*	*Female*	*45.5*	*54.5*	

**Table 8:** Case report: Fraction of overweight female and male patients for whom weight loss was thought to be important/not important by age and gender of GPs.

	Weight loss				
GPs age	N	Patients gender	Not important	Important	p
**≥57 years (male GPs)**	*332*				0.64
	*163*	*Male*	*38.7*	*61.3*	
	*169*	*Female*	*42.6*	*57.4*	
**≤56 years (male GPs)**	*294*				*0.004*
	*126*	*Male*	*28.6*	*71.4*	
	*168*	*Female*	*45.2*	*54.8*	
**≥49 years (female GPs)**	*235*				*0.01*
	*126*	*Male*	*27.8*	*72.2*	
	*109*	*Female*	*45.0*	*55.0*	
**≤48 years (female GPs)**	*217*				*0.21*
	*93*	*Male*	*21.5*	*78.5*	
	*124*	*Female*	*29.0*	*71.0*	

## Discussion

The results from the case reports suggest that Danish GPs still seem to give different advice and treatment recommendations to overweight male and female patients, and in general more often treat their overweight male than female patients. These findings are in line with results from a Swedish study, where GPs more often offered treatment for obesity to obese male than female patients. Furthermore, 18 years previously, when asked exactly the same question, Danish GPs also more often recommended weight loss to overweight male than female patients, presenting with similar symptoms [16]. Also, type of treatment to overweight men and women differed, in that lipid lowering drugs were recommended more frequently to male than to female patients. There has been a major development in the use of lipid lowering drugs since 1994, where the first survey round examining attitudes and treatment recommendations among Danish GPs was conducted. In 1994 lipid lowering medicine was infrequently used, whereas today it is widely used, because of its proven benefits in primary and secondary prevention, combined with the relatively few side effects [18]. However, this increase in use does not explain why the GPs more often prescribe lipid lowering drugs to their male than to their female patients. On the other hand, when comparing the results from the survey in 1994 [19] with the present results, it seems that there has been a shift in the work of GPs, from more therapeutic towards more preventive efforts.

Interestingly, female GPs slightly more often than male GPs recommended lipid lowering medicine to their overweight patients. However, as suggested by the interaction analysis, this difference between male and female GPs in medicine prescription was not further associated with differences in gender of the patients. Given that physical health complications related to obesity, such as type 2 diabetes [2], [3] and cardiovascular disease [9], or psychological correlates such as depression, stress and tiredness [12] [13] may be relatively more severe for overweight and obese women than men, this finding is surprising. Furthermore, the results obtained from the case reports covertly including gender are in conflict with the GPs beliefs’, that they treat male and female patients with overweight alike, the current results would suggest that the GPs are not aware that they offer different treatment to overweight men and women.

### Study Limitations

We cannot exclude the possibility that the GPs participating in the survey were those most motivated for prevention, and that some practitioners may have chosen not to participate because they found the topic to be of little relevance to them. Another option is that some GPs chose not to participate because of the length of the questionnaire. Indeed, some of the participating physicians expressed that they felt it was very time consuming to participate. However, it is unlikely that such bias in participation influenced the present results in relation to gender dependent treatment differences. It may also be argued that since only less than a third of all Danish GPs chose to participate in the survey, generalization of our results may be limited. However, there is no reason to assume that the observed differences in the *associations* between the recommendations for treatment of overweight among male and female patients cannot be generalized to all Danish GPs.

On the other hand, it may be difficult to generalize the results of the present study to GPs in other countries, where attitudes may be different, and where, among other things, culture and type of healthcare systems may influence the results. That being said, the fact that similar findings have been reported in studies from other countries [19] suggests that these findings may not be exclusive to Danish GPs.

## Conclusion

The GPs in the present study, where treatment practices towards male and female overweight patients were covertly measured, reported weight loss as treatment more often for overweight male than overweight female patients, presenting with the same mild symptoms. In addition, they also reported that they treated complications related to overweight more rigorously among overweight male than female patients, as they recommended lipid lowering medicine more often to male patients.

Given that such gender dependent treatment differences exist, our results call for studies on treatment differences between obese male and female patients.
